# ExEmPLAR (Extracting, Exploring, and Embedding Pathways Leading to Actionable Research): a user-friendly interface for knowledge graph mining

**DOI:** 10.1093/bioinformatics/btad779

**Published:** 2024-01-04

**Authors:** Jon-Michael T Beasley, Daniel R Korn, Nyssa N Tucker, Erick T M Alves, Eugene N Muratov, Chris Bizon, Alexander Tropsha

**Affiliations:** Division of Chemical Biology and Medicinal Chemistry, UNC Eshelman School of Pharmacy, University of North Carolina at Chapel Hill, Chapel Hill, NC 27599, USA; Department of Computer Science, University of North Carolina at Chapel Hill, Chapel Hill, NC 27599, USA; Division of Chemical Biology and Medicinal Chemistry, UNC Eshelman School of Pharmacy, University of North Carolina at Chapel Hill, Chapel Hill, NC 27599, USA; Department of Pharmacy, University of São Paulo, São Paulo, SP 05508, Brazil; Division of Chemical Biology and Medicinal Chemistry, UNC Eshelman School of Pharmacy, University of North Carolina at Chapel Hill, Chapel Hill, NC 27599, USA; Renaissance Computing Institute, University of North Carolina at Chapel Hill, Chapel Hill, NC 27599, USA; Division of Chemical Biology and Medicinal Chemistry, UNC Eshelman School of Pharmacy, University of North Carolina at Chapel Hill, Chapel Hill, NC 27599, USA; Renaissance Computing Institute, University of North Carolina at Chapel Hill, Chapel Hill, NC 27599, USA

## Abstract

**Summary:**

Knowledge graphs are being increasingly used in biomedical research to link large amounts of heterogenous data and facilitate reasoning across diverse knowledge sources. Wider adoption and exploration of knowledge graphs in the biomedical research community is limited by requirements to understand the underlying graph structure in terms of entity types and relationships, represented as nodes and edges, respectively, and learn specialized query languages for graph mining and exploration. We have developed a user-friendly interface dubbed ExEmPLAR (Extracting, Exploring, and Embedding Pathways Leading to Actionable Research) to aid reasoning over biomedical knowledge graphs and assist with data-driven research and hypothesis generation. We explain the key functionalities of ExEmPLAR and demonstrate its use with a case study considering the relationship of *Trypanosoma cruzi*, the etiological agent of Chagas disease, to frequently associated cardiovascular conditions.

**Availability and implementation:**

ExEmPLAR is freely accessible at https://www.exemplar.mml.unc.edu/. For code and instructions for the using the application, see: https://github.com/beasleyjonm/AOP-COP-Path-Extractor.

## 1 Introduction

Recent advances in high-throughput experimental techniques have led to an explosion of biological and chemical data, creating a critical challenge of intelligent data integration, harmonization, and efficient mining. Large-scale efforts to integrate existing multi-scale data sources, such as the NCATS Biomedical Data Translator program, seek to eliminate “data silos” and interlink a collective cross-disciplinary knowledge to enhance our understanding of human diseases and treatments (Austin *et al.* 2019). A critical tool for the Translator program is the use of information networks in the form of biomedical knowledge graphs (KGs), such as Reasoning Over Biomedical Objects linked in Knowledge Oriented Pathways (ROBOKOP) (Bizon *et al.* 2019). Biomedical KGs provide an efficient way to propose mechanistic explanations for drug therapeutic effect and/or chemical toxicity by representing facts involving biomedical concepts, such as drugs, proteins, and diseases, as semantic triples (subject, predicate, object) linked in graph databases. Indeed, the use of KGs has led to exciting and impactful research in recent times (Richardson *et al.* 2020, Bobrowski *et al.* 2021, Korn *et al.* 2021).

Effective reasoning over knowledge graphs requires knowledge of underlying graph structure, capacity to rapidly implement and tune queries, and tools to analyze numerous answers and substantiate inferences by examining primary knowledge sources. To address these needs, we have developed ExEmPLAR (Extracting, Exploring and Embedding Pathways Leading to Actionable Research), a web-based interface for mining knowledge graphs and embedding answer subgraphs for machine learning predictions.

## 2 Description

### 2.1 Biomedical knowledge graph sources

We developed ExEmPLAR based on the Neo4j graph database platform (https://neo4j.com/). The tool is designed to operate on knowledge graphs implemented in Neo4j such that any new Neo4j knowledge graph could be added with minimal development. Biomedical KGs currently implemented in ExEmPLAR include: ROBOKOP KG (Bizon *et al.* 2019), Hetionet (Himmelstein and Baranzini 2015), and CompToxAI (Romano *et al.* 2022).

### 2.2 Query construction tool

The primary functionality of ExEmPLAR is a graphical user interface (GUI) for rapidly constructing and editing queries in the Cypher query language and executing those queries on knowledge graphs linked to Neo4j databases. The ExEmPLAR interface allows users to construct queries that traverse a KG from a specified Start Node type to a specified End Node type. Users may construct up to 10 unique paths (P1–P10), with each individual path comprising up to 5 intermediate nodes (Levels 1–5). Start, End, and Level 1–5 nodes all include a text box for users to define specific node names or identifiers that must be present in answers. In addition to defining node types and entities, users may also define specific predicates between nodes to further specify searches.

### 2.3 Node search function

For user convenience, ExEmPLAR includes a function to search the selected KG for nodes names and IDs. Users can type partial or full node names or IDs in the “Starting Points” or “Ending Points” text boxes and search for nodes of the defined type which contain the searched string. Suggestions for node names and IDs will be displayed and can be copied into the search box for use in queries.

### 2.4 Answer table and visualization

Answers appear in tabular form below the query construction interface following retrieval from the KG. Each row in the answer table represents a single, unique answer subgraph. Columns can be hidden/unhidden by preference with the “Toggle Columns” button. The table can be downloaded by clicking the “Export” button. When the “Get Result MetaData” checkbox is selected, the text of node and edge properties can be viewed by hovering over the node or edge name with the mouse cursor. To visualize individual answer subgraphs, users may select the checkbox on the answer rows. Multiple answer rows, or all answers, can be added to build out a larger network based on a selected subset of answers. This function helps highlight critical answers and can aid hypothesis communication.

### 2.5 Save/load application state

ExEmPLAR includes a function to reproduce and share application settings and results by producing a downloadable file that encodes the current state of the application. The application state can be reloaded by uploading the file.

### 2.6 Ranking by PubMed abstract co-mentions

Due to the highly interconnected nature of biomedical KGs, longer query paths tend to return numerous answer subgraphs. ExEmPLAR can rank answers based on the number of abstracts available on PubMed (https://pubmed.ncbi.nlm.nih.gov/), which co-mention node names from KG answers.

When two columns are selected, only the counts between terms in the columns are returned. When three columns are selected, four abstract counts are returned: **node(A)–node(B)** counts, **node(A)–node(C)** counts, **node(B)–node(C)** counts, and the counts co-mention **node(A)**, **node(B),** and **node(C)**. In addition to count values, ExEmPLAR also creates columns hidden by default linking to the relevant PubMed co-mentioning abstracts.

ExEmPLAR’s ranking system is highly tunable to user needs. For instance, the user can choose to prioritize either well-known or under-described relationships between nodes depending on the context (e.g. prioritizing strongly supported relationships with numerous co-mentions or under-explored relationships with few co-mentions). Returning co-mention counts for three columns provides the additional benefit of allowing the user to “triangulate” support between the nodes in the columns. For example, when co-mentions exist for **node(A)**–**node(B)**, **node(A)**–**node(C)**, and **node(B)**–**node(C)** pairs, but no co-mentions exist for the **node(A)**–**node(B)**–**node(C)** triplet, one could infer that the individual facts between any two of **A, B,** and **C** are understood, but no known mechanism or hypothesis exists that encompasses all three nodes. (Swanson 1986). Recently, we used this method in combination with the ROBOKOP KG to explore biological mechanisms behind metal implant toxicity (Beasley *et al.* 2022). To improve specificity of the PubMed co-mention search, ExEmPLAR includes a function to convert gene symbols to the corresponding protein name according to the HUGO Gene Nomenclature Committee (Tweedie *et al.* 2021).

### 2.7 Answer embeddings and principal component clustering

Due to the highly interlinked nature of biomedical KGs, a highly connected node may interfere with valid novel hypothesis generation. A degree-weighted path count (DWPC) embedding for Start-End node pairs can be generated from the ExEmPLAR answer table. DWPC embeds the count of each metapath, or specific sequence of node and edge types between start and end nodes, and down-weights the contribution of paths through highly connected nodes. The details of the DWPC algorithm have been described previously (Himmelstein and Baranzini 2015) and machine learning using DWPC features been applied to drug repurposing (Himmelstein *et al.* 2017) and Alzheimer’s disease risk factor gene prediction (Binder *et al.* 2022). Users may visualize the proximity of Start-End pairs to one another in DWPC space by generating 2D and 3D scatter plots along the 2D and 3D principal components of DWPC features.

## 3 Case study

The protozoan parasite *Trypanosoma cruzi* (*T.cruzi*) is the etiological agent for Chagas Disease, which kills ten thousand people annually and affects nearly 7 million people worldwide, primarily in low-income communities in Latin-America. When the infection is not treated properly, the condition may progress to a chronic disease state wherein up to 30% of chronically infected people are prone to develop cardiac alterations and 10% can experience enlargement of gastrointestinal organs (PAHO/WHO 2023).

We demonstrated the utility of ExEmPLAR by examining paths through the ROBOKOP KG that may explain the mechanistic relationship between *T.cruzi* infection and heart conditions.

First, we constructed a query to ask which diseases are connected to the *T.cruzi* organism. *(OrganismTaxon(T.cruzi)—Disease)*

Querying ROBOKOP for direct associations in infection and disease returned results confirming that *T.cruzi* infection is correlated with the several heart conditions: cardiomyopathy, Chagas cardiomyopathy, myocarditis, dilated cardiomyopathy, hypertrophic cardiomyopathy.

We then constructed a query to ask which genes associated with the above cardiomyopathies are involved in biological processes or activities impacted by Chagas disease: *(OrganismTaxon(T.cruzi)-[causes]-Disease(Chagas disease)—Gene-[genetically_associated_with]-Disease(Heart conditions listed above))*

At the time of this writing, querying ROBOKOP returned 59 results from this search. From these results, select answer pathways were chosen to provide mechanistic insight into the relationship between Chagas disease and heart conditions.

**Figure 1.**
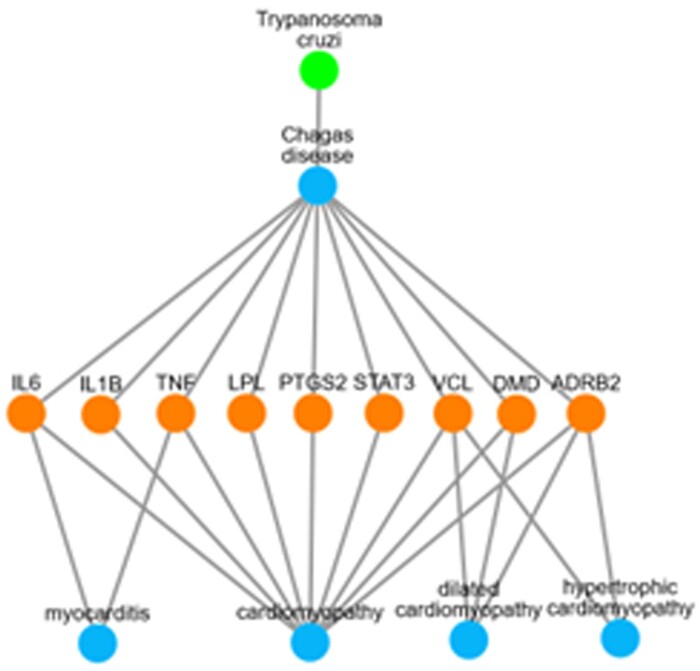
Chagas disease resulting from *T.cruzi* infection is associated with heart conditions including cardiomyopathy and myocarditis. ROBOKOP KG as analyzed by ExEmPLAR reveals a mechanistic pathway whereby *T.cruzi* infection may contribute to heart conditions via dysregulation of common genetic factors.

The results from these queries are summarized in Fig. 1, which is generated in ExEmPLAR using the row-wise network builder function. Figure 1 illustrates the causative agent *T.cruzi* leading to Chagas disease, and common genetic factors shared between Chagas disease and heart disorders.

As a response to *T.cruzi* infection and development of Chagas disease, a cascade of cytokines, such as IL-6, IL-β, (Savino *et al.* 2007), and TNF (Pereira *et al.* 2014), are upregulated to induce inflammation and activate lymphocyte cells to fight against the parasite. LPL and STAT3 have also been linked with the immune response to *T.cruzi* infection (Fu 2006, Chang 2019), and are related to cardiomyopathy, as shown in Fig. 1.

In addition, the figure suggests that Chagas disease can also be linked to cardiomyopathy through common associated genes, such as DMD, VCL, and ADRB2. (Nigro *et al.* 1994, Kamdar and Garry 2016, Deacon *et al.* 2019)

This case study can be readily reproduced by loading the state of the app from the “Trypanosoma_exemplar.pickle” file in Supplementary Information. It is important to note that changes to underlying knowledge graphs queried by ExEmPLAR may lead to different results if the query is repeated in the future.

## 4 Conclusions

We have developed ExEmPLAR graphical user interface (GUI) for biomedical KGs to enable members of the biomedical research community to rapidly engage with biomedical knowledge graphs. ExEmPLAR has been implemented as both webtool (https://www.exemplar.mml.unc.edu/) and standalone codebase (https://github.com/beasleyjonm/AOP-COP-Path-Extractor). This software addresses a critical issue standing in the way of wider adoption of knowledge graphs for biomedical study—the requirement to learn specialized query language skills and underlying KG structure.

With ExEmPLAR, users can (i) rapidly construct and tune KG queries, (ii) rank answer paths by PubMed abstracts that co-mention specific terms, (iii) pursue intriguing results by examining term co-mentioning links in the primary literature, (iv) visualize key hypothetical paths, and (v) ultimately, generate annotations of answer path features to group node pairs or train machine learning models in secondary workflows. Results and findings can be easily communicated and reproduced with the save/load application state feature.

## Supplementary Material

btad779_Supplementary_Data

## Data Availability

ExEmPLAR is freely accessible at https://www.exemplar.mml.unc.edu/. For code and instructions for the using the application, see: https://github.com/beasleyjonm/AOP-COP-Path-Extractor. This case study can be readily reproduced by loading the state of the app from the “Trypanosoma_exemplar.pickle” file in Supplementary Information, and ExEmPLAR has been implemented as both webtool (https://www.exemplar.mml.unc.edu/) and standalone codebase (https://github.com/beasleyjonm/AOP-COP-Path-Extractor).

## References

[btad779-B1] Austin CP , Colvis CM, Southall NT. Deconstructing the translational tower of babel. Clin Transl Sci 2019;12:85. 30412342 10.1111/cts.12595 PMC6440561

[btad779-B2] Beasley J-MT , Korn DR, Popov KI et al Integrated approach to elucidate metal-implant related adverse outcome pathways. Regul Toxicol Pharmacol 2022;136:105277. 36288772 10.1016/j.yrtph.2022.105277

[btad779-B3] Binder J , Ursu O, Bologa C et al Machine learning prediction and tau-based screening identifies potential Alzheimer’s disease genes relevant to immunity. Commun Biol 2022;5:125. 35149761 10.1038/s42003-022-03068-7 PMC8837797

[btad779-B4] Bizon C , Cox S, Balhoff J et al ROBOKOP KG and KGB: integrated knowledge graphs from federated sources. J Chem Inf Model 2019;59:4968–73. 31769676 10.1021/acs.jcim.9b00683 PMC11646564

[btad779-B5] Bobrowski T , Chen L, Eastman RT et al Synergistic and antagonistic drug combinations against SARS-CoV-2. Mol Ther 2021;29:873–85. 33333292 10.1016/j.ymthe.2020.12.016 PMC7834738

[btad779-B6] Chang CL. Lipoprotein lipase: new roles for an ‘old’ enzyme. Curr Opin Clin Nutr Metab Care 2019;22:111–5. 30648986 10.1097/MCO.0000000000000536 PMC6355338

[btad779-B8] Deacon DC , Happe CL, Chen C et al Combinatorial interactions of genetic variants in human cardiomyopathy. Nat Biomed Eng 2019;3:147–57. 30923642 10.1038/s41551-019-0348-9 PMC6433174

[btad779-B9] Fu XY. STAT3 in immune responses and inflammatory bowel diseases. Cell Res 2006;16:214–9. 16474436 10.1038/sj.cr.7310029

[btad779-B10] Himmelstein DS , Baranzini SE. Heterogeneous network edge prediction: a data integration approach to prioritize disease-associated genes. PLoS Comput Biol 2015;11:e1004259. 26158728 10.1371/journal.pcbi.1004259 PMC4497619

[btad779-B11] Himmelstein DS , Lizee A, Hessler C et al Systematic integration of biomedical knowledge prioritizes drugs for repurposing. eLife 2017;6:e26726. 28936969 10.7554/eLife.26726 PMC5640425

[btad779-B12] Kamdar F , Garry DJ. Dystrophin-deficient cardiomyopathy. J Am College Cardiol 2016;67:2533–46. 10.1016/j.jacc.2016.02.081 27230049

[btad779-B14] Korn D , Bobrowski T, Li M et al COVID-KOP: integrating emerging COVID-19 data with the ROBOKOP database. Bioinformatics 2021;37:586–7. 33175089 10.1093/bioinformatics/btaa718 PMC7890668

[btad779-B17] Nigro G , Politano L, Nigro V et al Mutation of dystrophin gene and cardiomyopathy. Neuromuscul Disord 1994;4:371–9. 7981594 10.1016/0960-8966(94)90073-6

[btad779-B18] PAHO/WHO. 2023. *Chagas Disease*. PAHO/WHO. Washington, D.C., USA: Pan American Health Organization. https://www.paho.org/en/topics/chagas-disease.

[btad779-B20] Pereira IR , Vilar-Pereira G, Silva AA et al Tumor necrosis factor is a therapeutic target for immunological unbalance and cardiac abnormalities in chronic experimental Chagas' heart disease. Mediators Inflamm 2014;2014:798078. 25140115 10.1155/2014/798078 PMC4130030

[btad779-B21] Richardson P , Griffin I, Tucker C et al Baricitinib as potential treatment for 2019-nCoV acute respiratory disease. Lancet 2020;395:e30–1. 32032529 10.1016/S0140-6736(20)30304-4 PMC7137985

[btad779-B22] Romano JD , Hao Y, Moore JH et al Automating predictive toxicology using ComptoxAI. Chem Res Toxicol 2022;35:1370–82. 35819939 10.1021/acs.chemrestox.2c00074 PMC9805296

[btad779-B23] Savino W , Villa-Verde DM, Mendes-da-Cruz DA et al Cytokines and cell adhesion receptors in the regulation of immunity to *Trypanosoma cruzi*. Cytokine Growth Factor Rev 2007;18:107–24. 17339126 10.1016/j.cytogfr.2007.01.010

[btad779-B24] Swanson DR. Fish oil, Raynaud’s syndrome, and undiscovered public knowledge. Perspect Biol Med 1986;30:7–18. 3797213 10.1353/pbm.1986.0087

[btad779-B27] Tweedie S , Braschi B, Gray K et al Genenames.org: the HGNC and VGNC resources in 2021. Nucleic Acids Res 2021;49:D939–46. 33152070 10.1093/nar/gkaa980 PMC7779007

